# Atomistic-geometry inspired structure-composition-property relations of hydrogen sII hydrates

**DOI:** 10.1038/s41598-023-46716-6

**Published:** 2023-11-11

**Authors:** Sahar Jafari Daghalian Sofla, Phillip Servio, Alejandro D. Rey

**Affiliations:** Department of Chemical Engineering, McGill University, Montreal, QC H3A 0C5 Canada

**Keywords:** Energy science and technology, Engineering, Materials science, Mathematics and computing

## Abstract

Gas hydrates are crystalline inclusion compounds formed by trapping gas molecules inside water cages at high pressures and low temperatures. Hydrates are promising materials for hydrogen storage, but their potential depends on understanding their mechanical properties. This work integrates density functional theory (DFT) simulations with a geometry-inspired composite material model to explore the bulk moduli of structure II hydrogen hydrates subjected to pressure loads of − 0.2 to 3 GPa, representative of the hydrogen hydrate formation conditions. Our findings reveal that structure II hydrate comprises a bi-continuous composite of small and large cages with nearly equal volume fractions. The bulk modulus increases with rising pressure but decreases with increasing composition. Notably, these results align closely with the ideal laws of mixtures, especially at low pressures and compositions, where cage interactions are minimal. This integrated DFT-laws of mixtures methodology provides a key database for fast estimation of hydrate mechanical properties without costly computations.

## Introduction

The world energy consumption has increased from 408 to 585 EJ with an average of 2% per year between 2000 and 2018 mainly due to the exponential population growth^1^. More than 80% of the world's energy demand is currently supplied with fossil fuels (e.g., oil, coal, natural gas), leading to the depletion of fossil fuels and environmental problems^2^. Fossil fuel combustion produces greenhouse gases that entail around three-quarters of the global greenhouse gas emissions^3^. Many countries have made a series of international and domestic climate change agreements, notably setting an ambitious target of “zero emissions’’ by 2050^4^. Striving for the shared objective of limiting global warming to 1.5 °C and a net zero emission goal requires a substantial transformation and transition across the energy sector^4^.

Hydrogen is viewed as the ‘fuel of the future’ and ‘green fuel’ since it has near zero emission and water vapor is the only product of combustion^5^. Hydrogen is the simplest and lightest element on Earth, which has remarkable properties such as high energy content per unit mass, approximately three times higher than that of natural gas^6^. Hydrogen’s higher energy conversion will save a huge amount of primary energy resource^1^. Also, the potential use of existing natural gas infrastructure for hydrogen transport makes hydrogen a more attractive alternative to fossil fuels^7^.

Hydrogen energy adaptation has faced many challenges, especially, the high cost of production (three times of petroleum products) demands a low-cost hydrogen storage system to circumvent the widespread use of this energy carrier^8^. Hydrogen storage remains the bottleneck of hydrogen vehicles and other applications due to limitations of current hydrogen storage systems in terms of capacity, safety, and cost, prompting the search for alternative technologies^5,8^. Among the emerging hydrogen storage techniques, the gas hydrate platform has shown immense potential for hydrogen storage. While current physical and chemical hydrogen storage systems possess volumetric capacities ranging from 0.013 to 0.039 kg/L^9^, volume capacities as high as 0.075 kg/L can be obtained by hydrates^10^. Gas hydrates are hydrogen-bonded crystalline solids with polyhedral structures that are capable of encapsulating guest gas molecules, such as hydrogen^11^. Specifically, the sII hydrate structure has demonstrated the capacity to store significant amounts of hydrogen^12^. Despite extensive research conducted on the thermodynamic properties and kinetics of sII hydrates^13–17^, there has been a noticeable lack of attention directed toward characterizing the mechanical properties of these structures. Consequently, the long-term hydrogen hydrate stability and mechanical strength under pressure loads and different compositions of the guest gas (i.e., hydrogen) are essentially nonexistent which is crucial for future progress in this area^15,16^^.^

Although primarily focused on methane hydrates rather than sII hydrates with hydrogen as the guest molecule, previous studies have used density functional theory (DFT) which has contributed to the understanding of hydrate mechanical properties. Zhu et al.^17^ used DFT and extensively characterized sI methane hydrates stability limits and the mechanical properties within the stability limits. Tensile and compressive stability limits of − 1 and 5.5 GPa were established from Born stability criteria, respectively. They conducted an extensive investigation into the piezo effect of sI methane hydrates across multiple scales, including atoms, cages, and lattice, which led to stability limits of − 1.1 and 7.5 GPa under 0 K^18^. They also successfully used laws of mixtures to confirm the elastic properties of sI methane hydrates. In another study, Zhu et al.^19^ investigated the failure mechanism for sI methane hydrates under pressure loads and found two different failure mechanisms depending on the occupancy of small and large cages. Jendi et al.^20^ also investigated the ideal strength bounds for sI methane hydrates and reported − 1.1 GPa and 90 GPa under tensile and compressive loads, respectively. Vlasic et al.^21^ examined the stability of sII hydrates with various guest gas molecules using the equation of state (EOS). The calculated bulk moduli values then were correlated with hydrogen bond density which is concluded to be the dominating force of the structure under compression. They found that the bulk moduli decrease as the size of the guest gas molecule increases leading to more compressible solids. Daghash et al.^22,23^ conducted an extensive analysis of sH hydrates, employing a first-principles method to fully characterize the physical and mechanical properties of sH hydrates. Their study quantified the influence of dispersion forces on the physical and mechanical properties of sH hydrates. By computing the elastic constants of sH hydrates under varying pressure conditions, they observed an increasing trend in elastic constants with pressure. Interestingly, they noted that different guest gas molecules exhibited distinct trends in elastic constants, further highlighting the importance of considering the specific properties of the guest molecules in sH hydrates. These previous contributions provide a solid ground to investigate and characterize sII hydrogen clathrates.

In this paper, we develop, implement, and validate an atomistic-geometry-inspired set of structure-composition-property relations. The structure is revealed by a representative lattice volume that captures an essential new feature for material properties. Along the structure, we find a crucial contribution of hydrogen occupancy, making chemical composition an important determinant of mechanical functionality. The properties we focus on in this nascent field emanate from Equation of State (EOS) that include compressibility and bulk modulus under pressure. The results from this work will be crucial in developing technologies for long-term hydrogen storage in hydrates. Although hydrates have distinct characteristics, the principles and methodologies used to derive geometry-composition-property relations can be applied to other material systems beyond hydrates. This includes material systems in various fields such as MTN-type zeolites which are known to have common microstructure as sII hydrate^24,25^, materials that have C15 Laves phase lattices such as metal hydrides^26^, or the ones that exhibit C15 Frank–Kasper-phases such as block-copolymers^27,28^.

## Results and discussion

### Structure analysis

**Figure 1:**
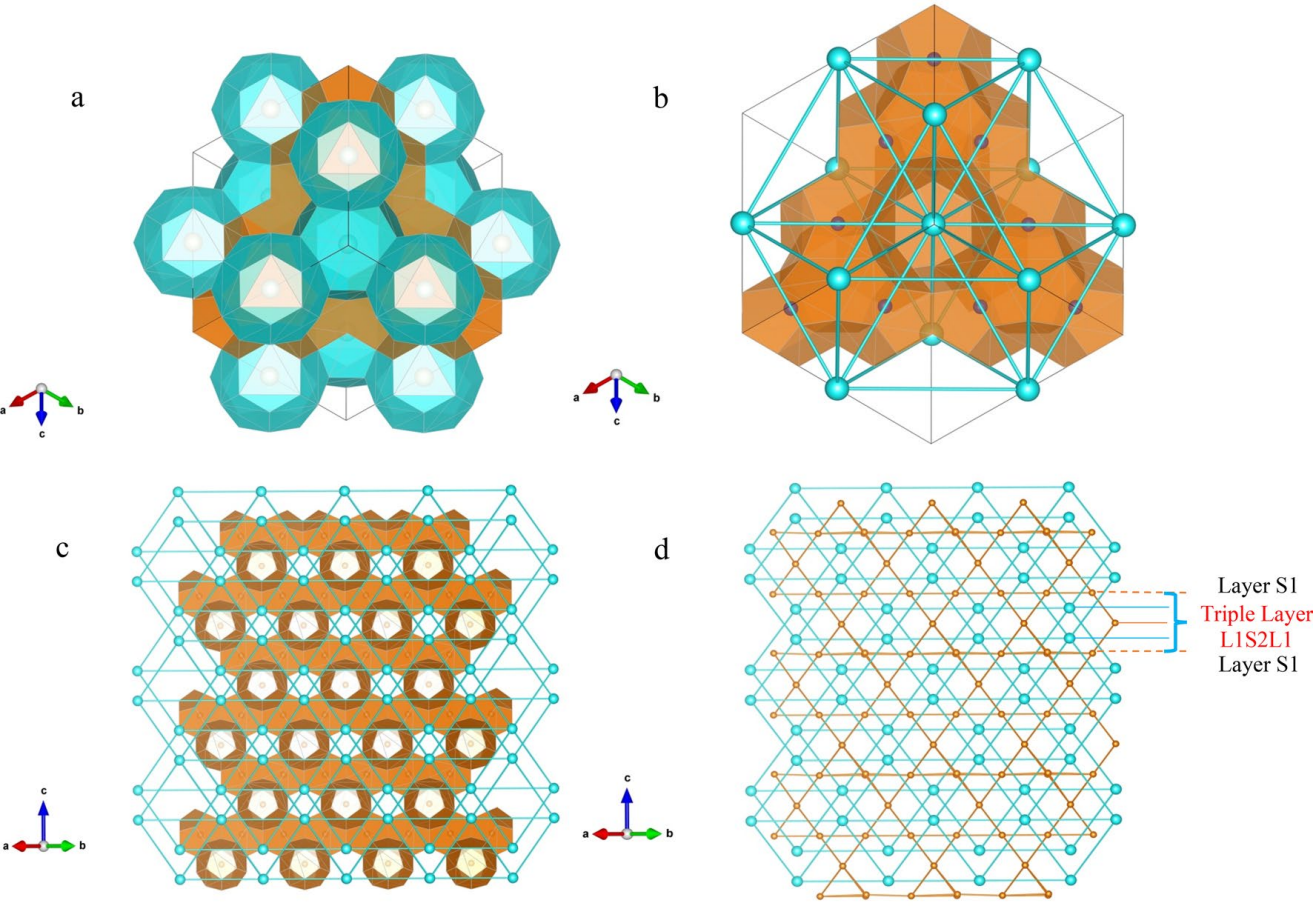
(**a**) The structure of sII lattice is represented by the interconnecting pyramids of small and large cages which is shown here in 3D. The small cages are shown in orange and large cages are shown in blue. (**b**) The connection between large cages in the lattice are shown by the blue nodes which correspond to the center of large cages. (**c**) The 2D structure of the sII hydrate is shown in [1 1 0] plane. (**d**) The connectivities between the cages is shown by connections of center of the cages. Orange nodes correspond to center of small cages while the blue nodes represent center of the large cages.

Structure II hydrate lattice has tetrahedral geometry which is comprised of 16 small cages and 8 large cages. Each large cage is surrounded by 4 large cages and 12 small cages while each small cage has 6 small and 6 large neighboring cages. For visualization, we used 3 dimensional visualization system for electronic and structure analysis (VESTA)^29^ displaying the multi-phase structure in Fig. 1. The unit cell structure is represented in 3D, and connections between the cages are highlighted by visualization of the center of small and large cages. While the top Fig. 1a,b illustrate the sII hydrate at the lattice scale, Fig. 1c,d provide an understanding of the connectivities between the cages in [1 1 0] plane of the bulk material. The structure of the sII hydrate at the lattice scale, can be broken down into interpenetrating pyramids of small and large cages as shown in Fig. 1a,b which was also observed by Chen et al.^30^. Alternatively, the stacked layer structure can be explained through a sequence of layers that follow the ABCB repeating pattern. The sequence ABCB for sII hydrates corresponds to alternating layers of small and large cages as S1L1S2L1 which is shown in Fig. 1d. We can see that both small and large cages at different layers are all connected to other large and small cages which proves the fact that sII hydrate has a bi-continuous structure.

The structural patterns found support the classification of sII hydrate as bi-continuous composite materials comprising small and large cages. This unique microstructure leads to distinct mechanical properties in sII hydrates, as the mechanical and physical characteristics of hydrates are influenced by their microstructural arrangement^31^. Unlike discontinuous or discrete composites that are reinforced by the inclusion of dispersed particles or fibers, the interpenetrating phases in bi-continuous materials can self-support their structures upon removal of either of the phases and behave like an open-cell and are known to generally have better mechanical properties due to enhanced load sharing and bending-dominated energy dissipation modes^31^.

The bi-continuity is also a very well-known concept in phase separation of polymers which can be achieved through spinodal decomposition that leads to two phases of polymer-rich and polymer-poor phases. Unlike nucleation and growth processes, spinodal decomposition occurs because of thermodynamic instability. The spinodal region is the unstable region in the free energy landscape and small fluctuations in the composition or density can lead to the collapse of the mixture into distinct phases^32^. However, sII structures are formed during a gradual process of nucleation and growth and are stable at their formation temperatures and pressures.

In sII hydrates, the volume fraction of both phases is close to 0.5 which means that we expect symmetric bi-continuous morphology, since there is no majority or minority component. By adopting a composite material approach, we can assess the individual effects of the small and large cages on the bulk modulus of the lattice. Comparisons between the results obtained from DFT simulations and the Murnaghan EOS, and the laws of mixtures enabled the accuracy validation of our computations and established robust structure-composition-property relations. As mentioned above, our findings build upon the work of Zhu et al.^18^, who investigated methane sI hydrates and validated the utility of the laws of mixtures in characterizing sI methane gas hydrates, with the crucial difference being that we have a bi-continuous composite instead of inclusion-matrix geometric structures.

### Bulk modulus

**Figure 2:**
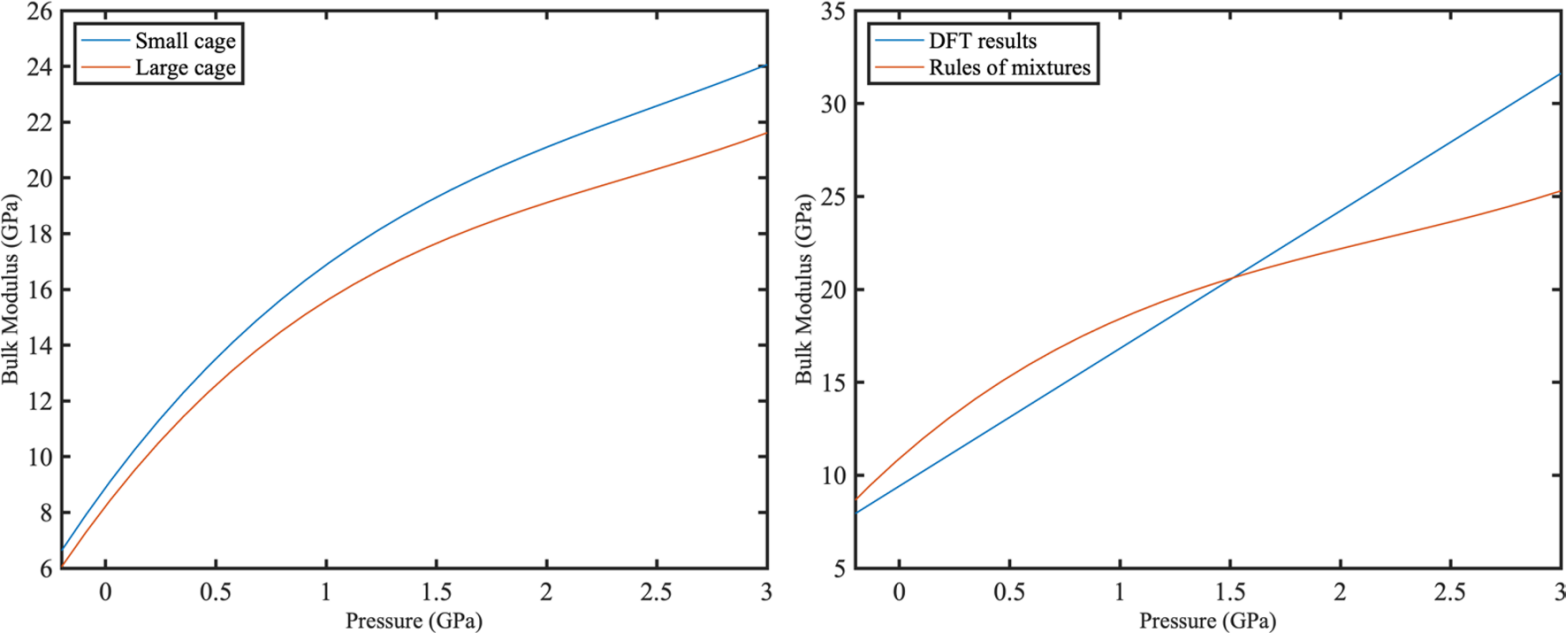
Averaged bulk modulus of small and large cages within − 0.2–3 GPa for an occupancy of 6 hydrogen molecules in the large cages and 2 hydrogen molecules in the small cages. The blue curve represents the averaged ${B}^{s}$ and the red curve represents the averaged ${B}^{l}$ (left). Bulk modulus of the lattice is calculated using two approaches. The blue curve represents the results obtained using DFT and Murnaghan EOS while red curve is obtained using laws of mixtures (right). The upper bound and lower bound of the bulk modulus from laws of mixtures overlaps for sII hydrogen hydrates.

Figure 2 demonstrates the bulk modulus of small and large cages for the case of 6 hydrogens in large and 2 hydrogens in the small cages within the pressure range of − 0.2–3 GPa. Figure 2 (left) illustrates the trend of the small and large cages' bulk moduli under pressure. It can be seen that both the small and large cages exhibit a consistent trend under pressure for the composition shown below and similarly, across all the other compositions examined in this study. The similar tendencies and values observed in the bulk modulus of the small and large cages under different occupancies and pressures can be attributed to the stress distribution in bi-continuous composites. The stress from applied pressure is evenly distributed throughout the continuous phases of the small and large cages. As a result, they exhibit comparable behaviors and values across different pressure and occupancy conditions.

The bulk moduli of the small and large cages increase with pressure and small cages are more rigid than the large cages which can be associated with their smaller size and shape. The fact that the small cage bulk modulus is larger than that of the large cage indicates that the large cages are more susceptible to deformations under tensile and compressive loads than the small cages at all the compositions. These results are consistent with those obtained by Zhu et al.^18^ who found the large cages to be more sensitive to size changes subjected to pressure loads than the small cages for sI methane hydrates. Figure 2 (right) shows that bulk moduli of the sII hydrate obtained from DFT and laws of the mixture are in good agreement at low pressures for the selected composition. The upper bound and lower bound of the bulk modulus from laws of mixtures overlap for sII hydrogen hydrates. The bulk modulus determined by the Murnaghan EOS, and the laws of mixtures come very close to each other when the pressure range is within − 0.2 to 1.5 GPa.

However, outside of this range, the bulk modulus predictions by the classical laws of mixtures start to diverge. This can be attributed to the increasing intermolecular forces between hydrogen molecules within the same cages, as well as their interactions with hydrogen molecules in adjacent cages, which is facilitated by shorter interatomic forces under compression. This, in turn, intensifies the interactions between the cages, which is not accounted for in ideal laws of mixtures.

**Figure 3:**
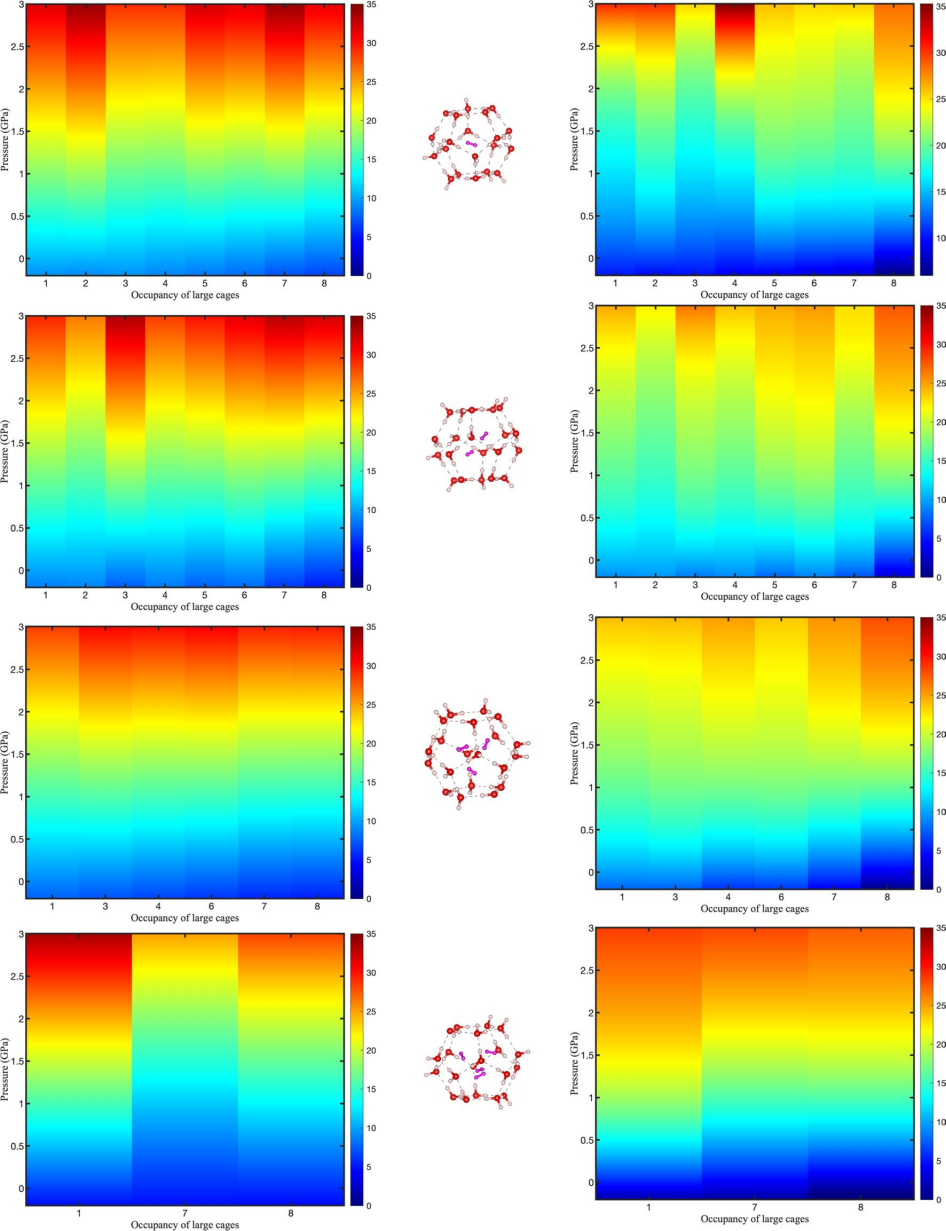
Diagrams of bulk modulus at different occupancy of small cages (**a**–**d**) with occupancy in the large cages varying between 1 and 8 from − 0.2 to 3 GPa. Red color corresponds to larger values of bulk moduli whereas blue corresponds to smaller values of bulk moduli with unit of GPa. Diagrams on the left column are obtained from DFT and Murnaghan EOS while diagrams on the right are obtained from laws of mixtures.

From the heat maps in Fig. 3, there is a strong positive correlation between the bulk modulus and the pressure for all studied compositions, ultimately reaching its maximum value at 3 GPa using the Murnaghan EOS. This shows that the structure becomes less compressible with increasing pressure. The trend of increasing piezo stiffness with an increase in pressure was also observed for methane and carbon dioxide hydrates^18,33^. It is evident that an increase in the hydrogen content of the large and small cages leads to a decrease in the bulk modulus and the structure becomes more compressible. From a quantitative perspective depicted in Fig. 3, it can be inferred that the influence of cage occupancy is relatively less significant within the pressure range of − 0.2 and 3 GPa as the variance of the bulk modulus is larger across the pressure range than the occupancy range. Furthermore, the higher hydrogen content of the small and large cages leads to a larger variance in bulk modulus values with pressure. The comprehension of the underlying reasons for these results lies within the supporting forces and the structure of the hydrogen hydrates.In hydrate structures, there are two supporting forces: host–guest interactions (van der Waals forces) and hydrogen bonding between host molecules. The dominant effect is primarily attributed to hydrogen bond densities at low pressures and occupancies, indicating that the host–guest interactions have not yet become significantly intensified and therefore do not exert a dominant influence^21^. However, as the pressure and occupancy increase, the effect of the guest molecule becomes more prominent which leads to comparatively lower compressibility. Although increasing the occupancy results in the expansion of cage volume^10^, which could potentially lead to more compressible structures because of the hydrogen bond elongation, the overall results do not show significant differences. This is because of the bi-continuous nature of the phases facilitating efficient stress transfer, mitigating the effects of cage expansion, and enhancing the mechanical properties. The load distribution between the small and large cages allows them to work together in a collective manner. The continuous bonding between the cages plays a crucial role in efficient stress transfer, contributing to the overall enhanced stability and mechanical properties. In other words, small and large cages reinforce each other, strengthening the structure.

The bulk modulus results obtained in this study are in the range of 8.20–14.56 GPa for various occupancies in the large cages and one hydrogen molecule in the small cages for compressive pressures between 0 and 0.5 GPa. This range decreases from 6.80 to 13.19 GPa when the small cage occupancy increases to two. For the cage occupancy of 3 or 4, the bulk moduli range from 6.86 to 12.25 GPa and from 5.76 to 11.01 GPa, respectively. These results are in accordance with the experimental findings of Manakov et al.^34^ at room temperature [(25 ± 5) °C] who found that the bulk modulus of gas hydrates is close to (9 $\pm \hspace{0.17em}$2) GPa for compressive pressures below 0.5 GPa for sI methane, sI xenon, and the sII double hydrate of tetrahydrofuran + xenon. The discrepancies can stem from the difference in the temperature, the nature of the guest gas molecule, and multiple occupancy of the cages. The bulk moduli at zero pressure also exhibit a range of variation, from 5.89 to 10.55 GPa, corresponding to the highest (8 hydrogen in the large cage and 4 in the small cages) and lowest (1 hydrogen in the large and 1 hydrogen in the small cages) studied compositions, respectively. Interestingly, these results closely align with the values obtained by Vlasic et al.^21^ for sII hydrate using a first principles-based method for hydrocarbon guest molecules. Specifically, the lowest compressibility observed in this study matches with the bulk modulus of 10.48 GPa obtained for the large cages solely occupied by propane by Vlasic et al.^21^.

**Figure 4:**
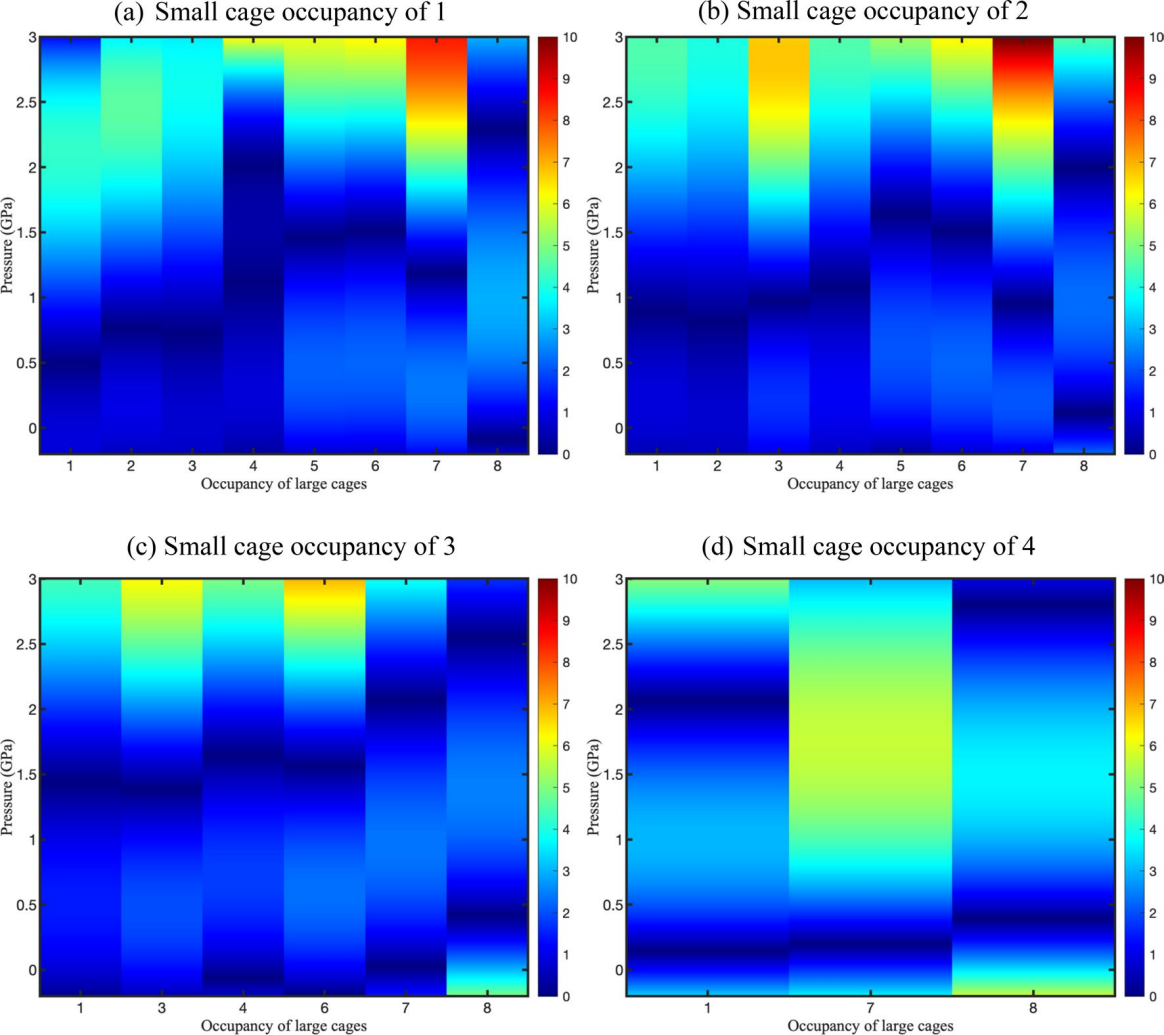
Diagrams show the discrepancy between bulk moduli obtained from DFT and laws of mixture within the pressure range of − 0.2 GPa and 3 GPa for small cage occupancy of 1, 2, 3, and 4. The blue color indicates pressures and occupancies where the results from the EOS and laws of mixtures lead to close values whereas the red color is where the difference is the maximum.

Figure 4 shows the difference between the bulk modulus calculated from DFT and laws of mixture. As can be seen, the difference between the results from the two methods is less than 2 GPa (or $\sim$ 25%) at most of the pressures and occupancies of the large cages when the small cages are occupied by one, two, or three hydrogens. This value drops below 1 GPa for the lower occupancies in the large cages which means that the laws of mixtures can be used to accurately predict the mechanical properties of hydrogen hydrates under certain conditions in which the cages do not interact strongly. However, the difference is more significant when the small cages are occupied by four hydrogen molecules. The interactions between the cages increase at this occupancy because the guest molecules become closer in different cages and interact more strongly. Since the cage interactions increase with increasing the number of hydrogen guests and pressure, the predictions of laws of mixture fail to fully capture this aspect. This was not seen in the sI methane hydrate studied by Zhu et al.^18^ since the occupancy of methane did not change and the composite had an inclusion-continuous structure. The increased interactions were confirmed during our previous study^10^, in which the hydrogen molecules in small and large cages started residing parallel to the pentagonal and hexagonal faces in small and large cages, diffusing to the neighboring cages. Once the occupancy in the small cages has reached 4, hydrogen molecules underwent translation parallel to pentagonal faces which led to hydrogen bond elongation, breakage, and collapse of the lattice^10^. For this reason, the hydrate structures are more compressible when the small cages are quadrupled occupied by hydrogen molecules compared to lower occupancies. Ideal laws of mixtures are not capable of accounting for the interaction of the cages as a result of hydrogen translation through the cages, which results in larger discrepancies in the predictions. It is also obvious from the derivative of bulk moduli at zero pressure as it increases with an increase in the number of hydrogen molecules in either large or small cages. This was also confirmed for sII hydrates with hydrocarbon guest molecules by Vlasic et al.^21^. They found that the ${B}_{0}^{\mathrm{^{\prime}}}$ (derivative of bulk modulus at zero pressure) is not constant and increases with the size of the guest molecule which signifies that hydrates get less compressible with increasing pressure. This value well exceeds 4 for hydrogen hydrates which is an accepted value for different gas hydrates^35–37^. This also confirms that the hydrates become less compressible with an increase in pressure due to the additional presence of hydrogen molecules inside the cages. As a result, we expect the bulk modulus of the hydrates to be more affected by the pressure at higher occupancies. Thus, the use of laws of the mixture to predict the mechanical properties of sII hydrates yields more accurate results under lower occupancy in small and large cages and low-pressure conditions.

## Conclusions

This paper investigated the effects of the geometry and the chemical composition of the small and large cages on the bulk moduli of sII hydrogen hydrates under pressure loads ranging from − 0.2 to 3 GPa. 3D visualization of the lattice revealed an interpenetrating bi-continuous composite-like structure of small and large cages. By using DFT in combination with the laws of mixtures, we found that small and large cages exhibit very close compressibility at tensile and low compressive pressures due to the negligible effects of the guest molecules at these pressures. However, as the compressive pressure increases, the small cages become less compressible compared to large cages across all occupancies. This suggests that small cages become stiffer with increasing pressure compared to the large cages. Both small and large cages, however, show similar tendencies under pressure and across various small and large cage occupancies, which is a direct manifestation of the bi-continuous structure. The bulk modulus of the lattice, obtained through DFT and the Murnaghan EOS, showed a linear positive correlation between compressibility and pressure across all the occupancies of small and large cages. Moreover, the bulk modulus values obtained from VASP and Murnaghan EOS are in excellent agreement with laws of mixture predictions under a wide range of pressure and various occupancies of small and large cages. The larger discrepancy at high pressures and high occupancies of the cages is found to be due to the increased interactions between the cages under these circumstances. While previous studies have investigated the bulk moduli of sII hydrates with various guest molecules, such as hydrocarbons and nitrogen^21,38^, the bulk modulus of sII hydrogen hydrates with different cage fillings and under a wide pressure range has not been studied before to the best of our knowledge. Hence, this study provided a comprehensive database on the bulk moduli of sII hydrogen hydrates.

## Methods

### DFT simulations

We used DFT as implemented in Vienna Ab initio Simulation Package (VASP)^39,40^ which is based on the Kohn–Sham equations. All parametric information for the DFT calculations is specified below. The output from VASP gives the ground state energy corresponding to the minimum energy of the atoms at different pressures and various occupancies at 0 K. In our simulations, we introduce a change in the lattice volume to generate a corresponding pressure. Then, the system is relaxed at a fixed volume of the cell (i.e., constant pressure) with two degrees of freedom (i.e., atomic positions).

To perform calculations of the energy in VASP, the initial position of the atoms in a unit cell should be supplied to the software. Then, VASP will apply the periodic boundary conditions to generate a supercell. sII hydrate has a cubic unit cell with 8 large cages and 16 small cages. The initial position of these cages is adapted from Takeuchi et al.^41^ who used X-ray diffraction to determine the position of the oxygen atoms. Next, they used Bernal–Fowler ice rules to determine the positions of the hydrogen atoms to minimize the dipole moment^42^. To generate the spatial position of hydrogen molecules inside the cages, we used Packmol software^43^ which is a geometric optimization tool. Packmol uses the “packing problem” to generate the initial positions for the atoms. In our simulations, all the cages have 100% occupancy and the occupancy of hydrogen in the small cages is varied from 1 to 4 while in the large cages, it is varied from 1 to 8 based on the occupancy limits established in our previous work^10^. Based on the defined tolerance, Packmol positions the specified number of hydrogen molecules inside a sphere (i.e., cages) with a specific center and radius. We provided Packmol with the empty sII hydrate positions and specified the number of hydrogen molecules we want within a radius from the center of the cages which are 3.91 nm and 4.73 nm for small and large cages, respectively^11,44^. The tolerance used for this work is 2.0 Angstroms. As a result, the structure relaxation was more efficient in terms of computational time due to the minimized net dipole moments.

After preparing the unit cell, rev-PBE exchange–correlation functional with DFT-D2 dispersion correction was used based on our previous work^17,18,21,22^. The Gamma-centered mesh with a size of 1 × 1 × 1 was selected based on the large face-centered cubic lattice of sII hydrate. For the electronic minimization, an energy cut-off of 520 eV was used which is 30% higher than the cut-off energy of the plane wave of oxygen atom based on the VASP manual recommendation. Force tolerance of 5 meV/Å was used for ionic relaxations. Projector augmented wave (PAW) potentials were used with a plane wave basis set^45,46^.

### Analysis

The generated energy-volume data leads to a function that is translated into a crystal EOS. The choice of the EOS was achieved based on the comparisons performed between the conventional EOSs for solids, including Murnaghan^47^, Birch-Murnaghan^48^, and Vinet^49^ and more details are provided in Supplementary Information 1 and Supplementary Data 1. The microstructural characteristics and effect of the geometry on the material bulk modulus were determined from the laws of mixture^18^. In our analysis, we used the ideal rules of mixtures, which provide upper and lower bounds for the properties depending on the direction of force being lateral or transverse. According to these two directions, the following relations are defined:

1 $$B_{upper} = \phi_{s} B_{s} + \phi_{l} B_{l}$$

and

2 $$B_{lower} = \left( {\frac{{\phi_{s} }}{{B_{s} }} + \frac{{\phi_{l} }}{{B_{l} }}} \right)^{ - 1}$$

where ${B}_{upper}$ and ${B}_{lower}$ are upper and lower bounds of bulk moduli, ${\upphi }_{s}$ and ${\upphi }_{l}$ stands for the volume fractions of the small and large cages, and ${B}_{s}$ and ${B}_{l}$ are the bulk moduli of small and large cages, respectively. Then, Voigt-Reuss-Hill (VRH) homogenization approximation is used to validate the results^50,51^. The following formula is used to calculate the bulk modulus of the constituents:

3 $$B_{i} = - V_{0}^{i} \frac{dP}{{dV^{i} }}; i = l,s$$

where ${B}_{i}$ is the bulk modulus of the “$i$” cage, $P$ is the pressure, and ${V}^{i}$ is the initial volume of the “$i$” cage, which is the volume at zero pressure in this work. $l$ and $s$ represent the large and small cages, respectively. This equation is applicable under constant temperature conditions, which is 0 Kelvin in this study. The volume of the cages can be determined from the Convhull method described in Supplementary Information 2, while the relationship between pressure and volume for each cage type was obtained by fitting a second-order polynomial to the data. The volume fraction of the small and large cages at each pressure and occupancy are calculated according to the following equations:

4 $$\phi^{s} = \frac{{16V_{avg}^{s} }}{{16V_{avg}^{s} + 8V_{avg}^{l} }}$$

5 $$\phi^{l} = \frac{{8V_{avg}^{l} }}{{16V_{avg}^{s} + 8V_{avg}^{l} }}$$

where ${\mathrm{V}}^{s}$ and ${\mathrm{V}}^{l}$ represents the volume fraction of small and large cages in a unit lattice. ${V}_{avg}^{s}$ shows the average volume of the 16 small cages and ${V}_{avg}^{l}$ is the average volume of the 8 large cages in a unit lattice.

### Supplementary Information

Supplementary Information.

## Data Availability

Data related to this work will be made available by request to the authors. These requests should be addressed to the corresponding author A.D.R. at alejandro.rey@mcgill.ca.
