# Supplementary material for: Atomistic-geometry inspired structure-composition-property relations of hydrogen sII hydrates
Source: Sci Rep. 2023 Nov 11;13:19675. doi: 10.1038/s41598-023-46716-6 (PMC10640630; doi:10.1038/s41598-023-46716-6)
Supplement: Supplementary file 1 — Supplementary Information. [file 41598_2023_46716_MOESM1_ESM.pdf]

Atomistic-Geometry Inspired Structure-Composition-Property Relations of Hydrogen sII Hydrates

Sahar Jafari Daghalian Sofla[1], Phillip Servio[1], & Alejandro D. Rey[1,*]

[1] Department of Chemical Engineering, McGill University, Montreal, Quebec, H3A 0C5, Canada

* Corresponding author: Alejandro D. Rey, Department of Chemical Engineering, McGill University, Montreal, Quebec, H3A 0C5, Canada.

Email: alejandro.rey@mcgill.ca

Tel: +1(514)3984400 Ext. 1026

Supplementary Information 1: Comparisons between different EOSs

The equation of state describes the relationship between temperature, pressure, and volume, such as the ideal gas law for gases. There is no such law (universal equation of state) for solid materials. However, there are many equations of state that include the material property like the Murnaghan equation [1]. There are three parameters needed for different equations of state, including 1) unit cell volume of the compound at zero pressure (V_0); 2) zero pressure bulk modulus (B_0) of the compound; 3) pressure derivative of bulk modulus (B_0') that can be calculated from the regression performed on the DFT generated energy-volume data. Murnaghan's equation of state assumes that the bulk modulus is a linear function of pressure:

$$B = B_0 + PB_0' \qquad \text{Eqn. S1}$$

which is only valid for low compression values of $0 < P < B_0/2$ because of the constant B'_0 assumption. Other two widely used equation of states for solids are Birch–Murnaghan equation [2], which is based on an expansion of the free energy in terms of the Eulerian strain f, where:

$$f = \frac{1}{2}\left[\left(\frac{V}{V_0}\right)^{\frac{-2}{3}} - 1\right] \qquad \text{Eqn. S2}$$

Another equation of state is the Vinet [3] equation, which is based on binding energy and length per electron. Both Birch-Murnaghan and Vinet EOSs assume that bulk modulus and first derivative of bulk modulus vary with pressure (is a function of pressure). Thus, the accuracy of each equation

of state depends on the pressure and volume range investigated. As the pressure range studied is not very wide, all three equations of state fit DFT data very well as the R^2=0.999 or higher is calculated from regression for all three EOSs. However, Murnaghan EOS gives slightly more accurate results which is used in bulk modulus calculations in this paper.

Below are the energy-explicit forms of the Murnaghan, Birch-Murnaghan, and Vinet EOSs, respectively.

$$\Delta E(V) = B_0 V_0 \left[\frac{1}{B_0'(B_0'-1)}\left(\frac{V}{V_0}\right)^{1-B_0'} + \frac{V}{B_0' V_0} - \frac{1}{B_0'-1}\right] \quad \text{Eqn. S3}$$

$$\Delta E(V) = \frac{9B_0V_0}{16}\left\{\left[\left(\frac{V_0}{V}\right)^{\frac{2}{3}} - 1\right]^3 B_0' + \left[\left(\frac{V_0}{V}\right)^{\frac{2}{3}} - 1\right]^2 \left[6 - 4\left(\frac{V_0}{V}\right)^{\frac{2}{3}}\right]\right\} \quad \text{Eqn. S4}$$

$$\Delta E(V) = \frac{4B_0V_0}{(B_0'-1)^2} - \frac{4B_0V_0}{(B_0'-1)^2}\left[1 - \frac{3}{2}(B_0'-1)\left(1 - \left(\frac{V}{V_0}\right)^{\frac{1}{3}}\right)\right] \exp\left[\frac{3}{2}(B_0'-1)\left(1 - \left(\frac{V}{V_0}\right)^{\frac{1}{3}}\right)\right] \quad \text{Eqn. S5}$$

The bulk modulus from VASP can be obtained by the following steps:

1) First, energy-volume curves are generated, which require relaxing the structure at different unit cell volumes to obtain the energy of the system at a specific volume.
2) Once the E-V data is obtained, the equations of state (EOSs) mentioned above can be fitted to assess their accuracy.
3) The accuracy of the EOSs in this paper is assessed using the R-squared value.
4) Murnaghan EOS was chosen because it provides the most accurate fit across all occupancies when compared to the Murnaghan-birth and Vinet EOSs. Then, E_0, V_0, and B_0' values are calculated using the EOS.
5) The pressure of the system can be calculated using the pressure definition at constant temperature (0 K), which is defined as:

$$P = \frac{dE}{dV} \quad \text{Eqn. S6}$$

$$P = \frac{B_0}{B_0'}\left[\left(\frac{V}{V_0}\right)^{B_0'} - 1\right] \quad \text{Eqn. S7}$$

6) Then, we construct pressure-volume curves at various occupancies of small and large cages.
7) Finally, the bulk modulus at various pressures can be calculated using the following formula:

$$B_{lattice} = -V_0^i \frac{dP}{dV^{lattice}};$$ Eqn. S8

where $B_{lattice}$ is the bulk modulus of the lattice P is the pressure and ${V_0}^i$ is the initial volume of the lattice, which represents the volume at zero pressure in this work.

Supplementary Information 2: Cage volume calculations

Small and large cages of the sII hydrate have polyhedral shapes, and the corresponding volumes can be approximated using the convex hull algorithm implemented in MATLAB. For a given set of vertices, the convex hull algorithm determines the smallest convex shape that encloses given set of coordinates. These vertices correspond to the coordinates of the oxygen atoms in the small and large cages which makes up the corners of the polyhedral shapes. To calculate the volume of the cages, the oxygen atoms of the 8 large and 16 small cages were identified. Then, the coordinates of the corresponding oxygen atoms were given to MATLAB. The "convhull" function of the MATLAB used to calculate the convex hull of the provided vertices. Below are the 3D plots of the shapes generated by "convhull" algorithm to calculate the corresponding volume of the large and small cages. Using this method, the lattice volume from VASP and from sum of all the cages were within 1% difference, which means that this method can be used reliably to calculate the volume of the hydrate cages.

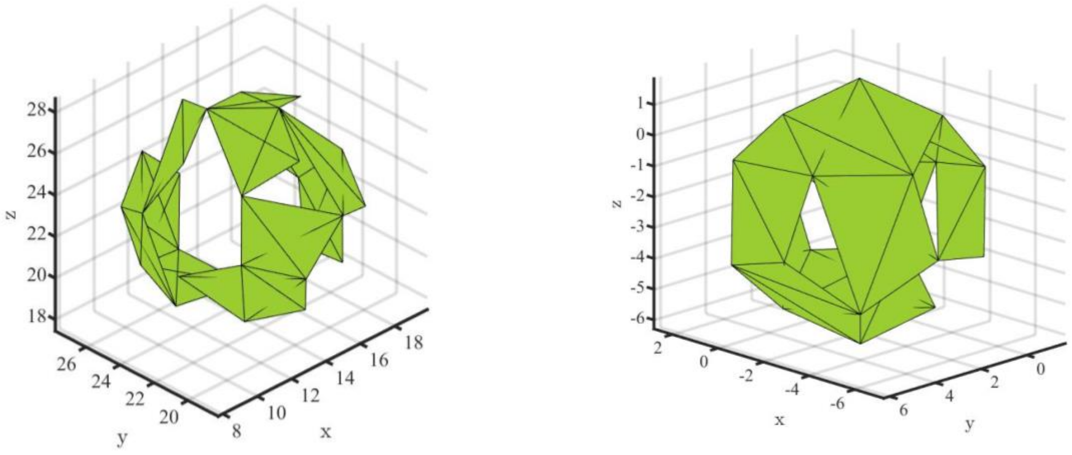

Supplementary Figure 1: The shapes generated by the Convex Hull algorithm to calculate the volume of the large and small cages.

Supplementary Data 1:

Tables below summarize the fitting parameters obtained from three EOSs for all the studied occupancies:

Occupancy												
EOS	L^1S^1			L^2S^1			L^3S^1			L^4S^1		
	V_0	B_0	B_0'	V_0	B_0	B_0'	V_0	B_0	B_0'	V_0	B_0	B_0'
Murnaghan	4912.57	10.55	5.87	4928.78	10.5	6.57	4965.62	10.25	6.11	5014.68	10.07	5.15
Birch-Murnaghan	4912.43	10.64	5.90	4928.12	10.64	6.68	4964.72	10.29	6.44	5013.09	10.19	5.41
Vinet	4912	10.55	5.87	4928	10.64	6.71	4965	10.29	6.48	5014	10.18	5.24

Occupancy												
EOS	L^5S^1			L^6S^1			L^7S^1			L^8S^1		
	V_0	B_0	B_0'	V_0	B_0	B_0'	V_0	B_0	B_0'	V_0	B_0	B_0'
Murnaghan	5069.31	9.64	5.94	5131.16	9.3	5.90	5221.04	8.51	6.91	5337.09	8.43	5.64
Birch-Murnaghan	5068.54	9.802	6.00	5130.16	9.54	5.84	5218.86	8.75	7.05	5337.15	8.21	6.51
Vinet	5068.35	9.83	6.02	5129.18	9.46	6.11	5220.97	8.72	6.92	5336.83	8.21	6.55

Occupancy

EOS	L^1S^2			L^2S^2			L^3S^2			L^4S^2		
	V_0	B_0	$B_0^'$	V_0	B_0	$B_0^'$	V_0	B_0	$B_0^'$	V_0	B_0	$B_0^'$
Murnaghan	5058.26	9.82	5.18	5085.2	9.82	4.29	5111.14	8.93	6.67	5151.38	9.52	4.96
Birch-Murnaghan	5057.38	9.9	5.31	5084.89	9.84	4.4	5109.29	8.96	7.28	5149.26	9.55	5.29
Vinet	5057	9.93	5.33	5085	9.86	4.47	5109	8.93	7.35	5148	9.56	5.41

Occupancy												
EOS	L^5S^2			L^6S^2			L^7S^2			L^8S^2		
	V_0	B_0	$B_0^'$	V_0	B_0	$B_0^'$	V_0	B_0	$B_0^'$	V_0	B_0	$B_0^'$
Murnaghan	5216.93	8.88	4.70	5279.95	8.71	5.29	5374.67	7.63	6.69	5504.47	7.07	6.42
Birch-Murnaghan	5215.9	8.94	4.82	5278.72	8.78	5.48	5371.32	7.85	6.94	5501.70	6.95	7.50
Vinet	5208.13	8.99	5.63	5277.66	8.79	5.63	5371.62	7.84	6.93	5501.98	6.97	7.37

Occupancy				
	L^1S^3	L^3S^3	L^4S^3	L^6S^3

EOS	V_0	B_0	B_0'	V_0	B_0	B_0'	V_0	B_0	B_0'	V_0	B_0	B_0'
Murnaghan	5256.62	8.87	6.00	5313.03	8.68	5.14	5358.97	8.09	5.68	5482.46	7.54	5.92
Birch-Murnaghan	5256.13	8.95	6.07	5311.42	8.72	5.39	5356.79	8.12	6.08	5478.10	7.66	6.31
Vinet	5261.4	8.89	5.28	5311.15	8.74	5.44	5356.27	8.12	6.16	5478.4	7.68	6.31

Occupancy															
EOS	L^7S^3			L^8S^3			L^1S^4			L^7S^4			L^8S^4		
	V_0	B_0	B_0'	V_0	B_0	B_0'	V_0	B_0	B_0'	V_0	B_0	B_0'	V_0	B_0	B_0'
Murnaghan	5587.08	7.34	5.72	5686.36	7.21	5.52	5521.47	6.67	6.93	5765.81	8.023	4.58	6010.51	4.94	6.89
Birch-Murnaghan	5583.73	7.30	6.41	5690.63	6.85	6.61	5517.58	6.58	8.19	5769.15	7.79	5.16	6017.79	4.41	9.61
Vinet	5583.4	7.30	6.46	5691.48	6.80	6.73	5518.88	6.56	7.99	5772.06	7.62	5.51	5989.9	5.541	6.7

References

1. Murnaghan, F. D. The Compressibility of Media under Extreme Pressures. *Proc. Natl. Acad. Sci.* **30**, 244–247 (1944).
2. Birch, F. Elasticity and constitution of the Earth's interior. *J. Geophys. Res. 1896-1977* **57**, 227–286 (1952).
3. Vinet, P., Ferrante, J., Rose, J. H. & Smith, J. R. Compressibility of solids. *J. Geophys. Res. Solid Earth* **92**, 9319–9325 (1987).
